# Composition-Dependent Mechanical and Thermal Behavior of TPU-Modified PLA and ABS Filaments for FDM Applications

**DOI:** 10.3390/polym18080949

**Published:** 2026-04-13

**Authors:** Burak Demirtas, Caglar Sevim, Munise Didem Demirbas

**Affiliations:** 1 Erciyes University, Graduate School of Natural and Applied Sciences, Department of Mechanical Engineering, Kayseri 38039, Türkiye; burakdemirtas@kayseri.edu.tr; 2 Department of Mechanical and Metal Technologies, Vocational College of Organized Industrial Zone, Welding Technology Program, Kayseri University, Kayseri 38170, Türkiye; 3 Department of Mechanical Engineering, Faculty of Engineering, Niğde Ömer Halisdemir University, Niğde 51240, Türkiye; caglar.sevim@ohu.edu.tr; 4 Department of Mechanical Engineering, Faculty of Engineering, Erciyes University, Kayseri 38280, Türkiye; 5 Aviation Research and Application Center, Erciyes University, Kayseri 38280, Türkiye; 6 Natilus Engineering, Erciyes Teknopark, Kayseri 38039, Türkiye

**Keywords:** PLA/TPU blends, ABS/TPU blends, thermoplastic polyurethane (TPU), material extrusion, digital image correlation (DIC), differential scanning calorimetry (DSC) introduction, additive manufacturing

## Abstract

Although polylactic acid (PLA) and acrylonitrile–butadiene–styrene (ABS) are among the most widely used polymers in material extrusion, their limited toughness and energy-absorption capacity often restrict the structural performance of 3D-printed functional components. To address the limited comparative understanding of how thermoplastic polyurethane (TPU) modifies the deformation behavior and phase characteristics of these two polymer systems, this study presents a multi-analytical evaluation of TPU-reinforced PLA and ABS blends. To this end, both polymers were blended with TPU at 10–50 wt% and processed into filaments via single-screw extrusion. The resulting filaments were used to fabricate ASTM D638 Type I tensile specimens via material extrusion under matrix-specific, but internally consistent, printing parameters. For each composition, five specimens were tested to obtain representative values of tensile strength, elongation at break, and toughness. In addition to conventional tensile testing, the evolution of strain during deformation was monitored using digital image correlation (DIC), enabling full-field characterization of local deformation behavior. To ensure experimental reliability, specimen masses were carefully controlled, and the datasets were analyzed using MATLAB. Thermal properties were investigated by differential scanning calorimetry (DSC) to determine the influence of TPU on glass transition, melting behavior, and phase mobility, and to relate these thermal characteristics to the mechanical response of the blends. The incorporation of TPU significantly increased ductility and energy absorption in both polymer matrices, although the magnitude of improvement differed. ABS/TPU blends exhibited the highest toughness enhancement, reaching 221.4% at 30 wt% TPU, while PLA/TPU systems showed nearly a twofold increase at 20 wt% TPU. DIC analysis further revealed a transition from localized brittle deformation in neat polymers to more distributed plastic deformation with increasing TPU content. DSC results indicated reduced crystallinity in PLA-rich blends and enhanced segmental mobility in ABS-based systems, consistent with the observed mechanical behavior. Overall, the combined mechanical, optical, and thermal analyses demonstrate that the optimal TPU content is matrix-dependent, providing practical guidelines for tailoring PLA- and ABS-based filaments to achieve a controlled balance between stiffness, ductility, and energy absorption in material extrusion applications.

## 1. Introduction

Additive manufacturing (AM), and particularly fused deposition modeling (FDM), has become a widely adopted approach for fabricating polymer components with controlled geometry at relatively low production cost [1]. Among commercially available feedstocks, polylactic acid (PLA) and acrylonitrile–butadiene–styrene (ABS) remain dominant due to their good processability and dimensional stability during printing [2]. However, neither material is ideal from a mechanical standpoint. PLA is inherently brittle and exhibits limited strain accommodation, whereas ABS, although tougher, often suffers from interlayer weakness and warpage, particularly under suboptimal thermal control during printing [3,4]. The inability of these neat polymers to withstand significant impact or cyclic loading remains a critical bottleneck, restricting their use in high-performance structural applications where durability is paramount. Consequently, this study provides a controlled, side-by-side evaluation to establish a reproducible framework for optimizing TPU-modified filaments. These limitations continue to restrict their broader structural applications.

Blending with thermoplastic polyurethane (TPU) has therefore attracted considerable attention as a practical toughening strategy. Owing to its segmented elastomeric structure, TPU introduces flexibility, impact resistance, and enhanced energy dissipation into otherwise rigid polymer matrices [5,6,7]. In PLA-based systems, improvements in elongation at break and fracture resistance have been repeatedly reported, particularly when compatibilization strategies such as chain extension or in situ copolymer formation are employed [8,9,10,11]. Recently, the development of glycidol-free aliphatic copolymers has emerged as a safer and more sustainable alternative to commercial epoxy-based chain extenders, effectively enhancing the molecular weight and mechanical performance of PLA matrices [12]. Additionally, the phase compatibility and flame retardancy of such systems have been explored to broaden their industrial utility [13]. Fang et al. demonstrated that incorporating PLA–TPU copolymers significantly increased tensile elongation while maintaining acceptable strength [1]. Similarly, Rahmatabadi et al. observed marked improvements in fracture toughness in FDM-processed PLA/TPU blends with increasing TPU content [11].

Comparable trends have also been reported for ABS-based blends. The addition of TPU reduces brittleness and enhances the energy absorption capacity relative to neat ABS [5,11,14]. Thavornyutikarn et al. further showed that ABS/TPU systems not only exhibit enhanced toughness but may also offer improved compatibility, extending their relevance beyond conventional structural applications [4]. These findings collectively indicate that TPU acts as an effective ductility modifier across chemically distinct thermoplastic matrices, although the underlying mechanisms differ. Thermal behavior provides important insight into these mechanisms. TPU incorporation is known to influence glass transition behavior and crystalline structure, often lowering the glass transition temperature and modifying melting characteristics in both PLA and ABS matrices [12,15,16]. Differential scanning calorimetry (DSC) has therefore been widely used to interpret changes in crystallinity, phase mobility, and miscibility in such blends [17,18]. At the same time, extrusion parameters—including screw speed, temperature profile, and additive dispersion—play a decisive role in determining filament homogeneity and the resulting mechanical consistency [19,20].

Beyond bulk mechanical measurements, deformation mapping techniques have become increasingly important. Digital image correlation (DIC) enables full-field strain analysis during tensile testing and captures localized deformation phenomena that conventional extensometry may overlook [21,22,23]. This approach is particularly valuable for FDM materials, where layer-induced anisotropy can significantly influence stress–strain evolution [14,24]. When combined with statistical variation analysis, DIC-based measurements can improve data robustness and reproducibility [25,26]. Moreover, response surface methodologies, such as the Box-Behnken design, have been effectively employed to assess the coupled effects of composition and printing parameters on the performance of PLA-TPU composites [27]. Recent research has also expanded toward multifunctional and shape-memory PLA/TPU and ABS/TPU systems [15,28,29]. For instance, Nejatpour et al. reported promising shape-memory recovery in PLA/TPU blends with stable thermal behavior [15], while Soltanmohammadi et al. systematically evaluated ABS/TPU blends and identified substantial toughness gains at moderate TPU loadings [5]. Additional strategies, including nanofiller incorporation [8,27], the use of flame retardants [13] and architected metamaterial designs [30,31], have further broadened the performance potential of these blends for energy-absorbing, biomedical, and adaptive applications. Furthermore, the role of TPU type and concentration in governing heat-triggered recovery behavior has been identified as a key design factor [32]. Despite this growing body of research, direct, systematic comparisons between PLA/TPU and ABS/TPU systems processed under unified extrusion and printing conditions remain limited. Most studies focus on a single matrix at a time, making cross-material interpretation difficult [1,5,11]. Moreover, only a limited number of investigations combine DSC, tensile testing, and full-field DIC within a single framework to explicitly relate thermal transitions to strain localization behavior [14,16,23]. Although TPU modification has been widely investigated as a toughening strategy for both PLA and ABS, most existing studies evaluate these matrices independently, making direct cross-material interpretation difficult. In addition, studies integrating thermal analysis, mechanical testing, and full-field strain mapping within a unified comparative framework remain scarce. Therefore, the novelty of the present work lies in its controlled, side-by-side evaluation of PLA/TPU and ABS/TPU blends processed under consistent extrusion and printing conditions.

In this context, the present study provides a controlled, side-by-side evaluation of PLA/TPU and ABS/TPU blends across a broad TPU content range (10–50 wt%). All materials were extruded under consistent processing conditions and characterized through tensile testing, DSC analysis, and full-field DIC. The tailored mechanical profiles of the developed TPU-modified filaments broaden their potential for use in functional FDM components that require a balance between structural rigidity and energy absorption. Specifically, PLA/TPU blends may be advantageous for relatively rigid yet impact-tolerant parts such as custom-fit protective covers and lightweight housings. In contrast, ABS/TPU systems may be more suitable for semi-flexible industrial components, such as vibration-damping mounts, resilient gaskets, and flexible coupling covers. These material systems therefore offer promising potential for the fabrication of machine-related components where reduced brittleness and improved deformation tolerance are desired. By incorporating specimen-level mass verification and statistical error analysis, this study aims to establish a reproducible and application-oriented framework for optimizing TPU-modified PLA and ABS filaments in advanced FDM manufacturing.

## 2. Materials and Methods

### 2.1. Materials

Commercial-grade polymers were used as received unless otherwise specified. ABS (HI121H, LG Chem Ltd., Seoul, Republic of Korea), PLA (Luminy^®^ LX175, Total Corbion PLA BV, Gorinchem, The Netherlands), and TPU (Ravathane^®^ 140 A90, Ravago Petrokimya, Kocaeli, Turkey) were selected for this study. Prior to processing, the polymer pellets were dried to minimize moisture-related effects during extrusion and printing. PLA and ABS pellets were dried at 85 °C for 4 h to achieve moisture levels below 250 ppm, while TPU pellets were dried at 90–105 °C for 2 h to maintain moisture content below 0.02%, in accordance with the supplier’s recommendations.

Key material properties were obtained directly from the manufacturers’ technical datasheets provided by LG Chem (ABS HI121H), Total Corbion (PLA Luminy^®^ LX175), and Ravago Petrokimya (Ravathane^®^ 140 A90 TPU) [24,33,34] and are summarized in Table 1.

### 2.2. Filament Extrusion, FDM 3D Printing, and Specimen Preparation

**Figure 1:**
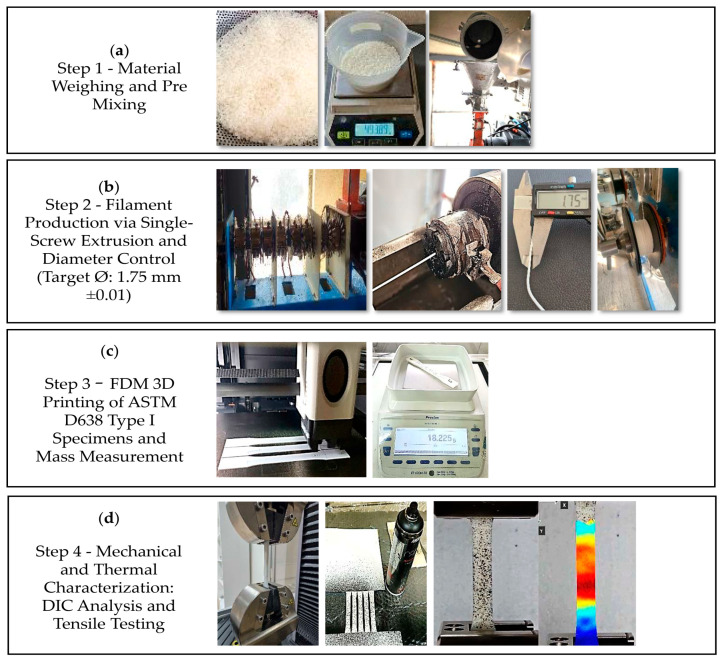
Schematic representation of the experimental process, including (**a**) material weighing and pre-mixing, (**b**) filament extrusion and diameter control, (**c**) 3D printing of ASTM D638 Type I specimens, and (**d**) mechanical and thermal characterization by tensile testing and DIC analysis.

Blend filaments were produced using a single-screw extrusion line equipped with a temperature-controlled heating zone and a synchronized haul-off unit, a method commonly applied in polymer blend preparation for FDM feedstock [8,21,22,27]. The overall experimental workflow, including material preparation, filament production, and specimen fabrication, is schematically illustrated in Figure 1. For the PLA/TPU blends, the screw rotation speed was maintained at 20 rpm with a haul-off speed of 21 m·min^−1^. Meanwhile, for the ABS/TPU blends, the screw rotation speed was set to 26.5 rpm with a haul-off speed of 28 m·min^−1^. This ensures stable throughput and consistent filament formation [20].

During extrusion, a Sikora LASER Series 2000 optical diameter gauge (Sikora AG, Bremen, Germany) continuously monitored the filament diameter in real time, similar to previous diameter-control techniques employed for high-performance polymer blends [23,35]. After production, the filament diameter was further verified using a digital caliper (accuracy ±0.01 mm) at multiple positions along each batch to confirm dimensional stability and uniformity. The extrusion parameters for PLA/TPU and ABS/TPU blends are summarized in Table 2.

Tensile test specimens based on ASTM D638 Type I [36] geometry were fabricated using a Bambu Lab P1S fused deposition modeling (FDM) printer equipped with a 0.4 mm stainless steel nozzle, consistent with recent studies that adopted a similar geometry for polymer blend evaluation [34,35]. Printing was carried out with a 100% concentric infill pattern to eliminate voids and ensure full material continuity across the gauge section, a strategy also applied to enhance repeatability in mechanical characterization [25,27]. Specimen thickness was intentionally increased to 6 mm in ASTM D638 Type I to improve the structural stability and repeatability of tensile measurements for the TPU-modified blends [7,11,37,38]. A constant layer height of 0.2 mm, print speed of 250 mm·s^−1^, and filament diameter of 1.75 mm were employed, which fall within the parameter range widely reported for high-strength FDM composites [21,23,39]. The nozzle and bed temperatures were optimized for each blend type: 220 °C/65 °C for PLA/TPU blends and 260 °C/95 °C for ABS/TPU blends, in agreement with the optimized conditions reported in recent extrusion-based studies [20,21,29]. The printing parameters are summarized in Table 3.

### 2.3. Tensile Testing and Mass Verification of 3D Printed Specimens

**Figure 2:**
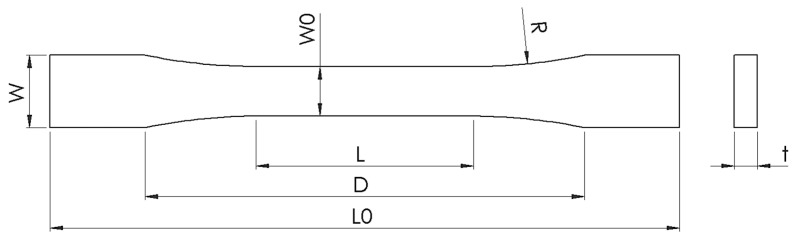
Geometry and dimensions of the ASTM D638 Type I tensile specimen used in this study, with the following parameters: W = 19 mm, W_0_ = 13 mm, t = 6 mm, L_0_ = 165 mm, L = 57 mm, and D = 115 mm.

Tensile behavior was evaluated using a universal testing machine (MTS Criterion, Eden Prairie, MN, USA) in accordance with ASTM D638 Type I. A crosshead speed of 0.02 mm s^−1^ was selected to ensure stable deformation and to facilitate high-resolution DIC measurements by limiting excessive speckle displacement between consecutive frames. For each blend composition, five specimens were tested under identical conditions. Tensile strength (MPa), elongation at break (%), and toughness (J/m^3^) were determined from the resulting stress–strain curves. The raw force (kN), displacement (mm), and time (s) data were processed in MATLAB to construct the stress–strain responses. Mean values, standard deviations, and coefficients of variation (CV) were calculated to evaluate experimental scatter [39]. Error-bar plots representing maximum stress values were generated from these datasets to enable consistent comparison across all compositions. To ensure dimensional consistency and compositional uniformity, each ASTM D638 Type I specimen (Figure 2) was weighed prior to mechanical testing using a high-precision analytical balance (EP 620M-FR, Precisa^®^, Dietikon, Switzerland; readability 0.001 g; maximum capacity 620 g). For every PLA/TPU and ABS/TPU blend ratio (10–50 wt% TPU), five specimens were fabricated and analyzed. The measured masses remained within ±1.5% of the corresponding group mean, indicating consistent filament extrusion and stable printing conditions. This specimen-level mass verification served as an additional quality-control measure supporting the reliability of the mechanical data.

### 2.4. Digital Image Correlation (DIC) Analysis

High-resolution video recordings (3840 × 2160, 60 fps) were captured using a tripod-mounted camera with a dedicated lighting system to ensure uniform illumination and minimize shadows during testing. DIC analysis was performed in MATLAB R2024a (The MathWorks Inc., Natick, MA, USA) using the Ncorr open-source DIC algorithm [40], while image processing procedures were conducted with MATLAB’s Image Processing Toolbox. To enable accurate strain mapping, a fine speckle pattern was applied to the gauge section of each specimen by spraying black paint onto the surface. This procedure was consistently applied to all blend compositions to ensure reliable correlation during the DIC analysis [20,22,23,40].

### 2.5. Differential Scanning Calorimetry (DSC) Analysis

The thermal behavior of the blend materials was characterized using a PerkinElmer STA 6000 differential scanning calorimeter (PerkinElmer Inc., Waltham, MA, USA). The instrument operates over a temperature range of 25–300 °C with programmable heating rates of 0.1–50 °C min^−1^, allowing simultaneous monitoring of thermal transitions and mass changes. Representative samples from each blend composition were analyzed under a nitrogen atmosphere using controlled heating conditions. The measurements were conducted to determine the glass transition temperature (Tg), melting temperature (Tm), and crystallization temperature (Tc), together with their corresponding enthalpy changes [33,34]. These parameters provided insight into thermal transitions, crystallinity evolution, and phase interactions in the PLA/TPU and ABS/TPU systems. To ensure material representativeness and consistent thermal analysis, DSC specimens were not directly cut from the extruded filaments. Instead, dedicated samples were 3D printed from each blend filament and subsequently sectioned into DSC-compatible specimens, minimizing potential variability associated with filament surface defects or irregular cross-sections. The resulting thermal data were used to support the mechanical characterization and to interpret the structure–property relationships observed in the blend systems [41,42,43].

## 3. Results

### 3.1. Specimen Mass Verification

Prior to mechanical testing, five ASTM D638 Type I specimens were weighed for each blend composition using a precision analytical balance. The mean mass values and corresponding standard deviations for both PLA/TPU and ABS/TPU formulations are summarized in Table 4.

The measured masses exhibited very low variability across all compositions, indicating consistent filament feeding and stable specimen fabrication during the FDM process. CV remained below 0.4% for all blends, demonstrating excellent repeatability and suggesting a negligible influence of material flow fluctuations during printing.

### 3.2. Tensile Strength and Statistical Analysis

**Figure 3:**
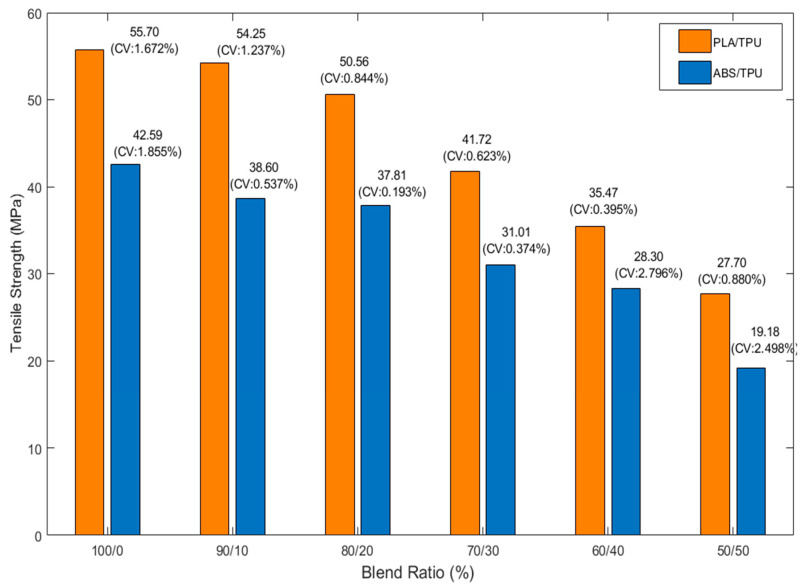
Maximum tensile strength values (mean ± SD) of PLA/TPU and ABS/TPU blends at various TPU blend ratios. Error bars represent standard deviation (*n* = 5). CV% values are also indicated to demonstrate the consistency of the mechanical test results.

Tensile strength values of PLA/TPU and ABS/TPU blends were determined for TPU contents ranging from 0 to 50 wt%. As shown in Figure 3, increasing TPU content resulted in a gradual reduction in maximum tensile stress for both material systems. For PLA-based blends, the tensile strength decreased from 55.70 MPa (CV = 1.672%) for neat PLA (0 wt% TPU) to 27.70 MPa (CV = 0.880%) for PLA at 50 wt% TPU. A similar trend was observed for ABS/TPU blends, where strength declined from 42.59 MPa (CV = 1.855%) to 19.18 MPa (CV = 2.498%) over the same composition range. At comparable TPU contents, PLA/TPU specimens consistently exhibited higher tensile strength than ABS/TPU specimens, reflecting the inherently higher stiffness of the PLA matrix. The standard deviation remained relatively small for all compositions, ranging from 0.140 to 0.932 MPa for PLA/TPU and 0.073 to 0.791 MPa for ABS/TPU blends. All CV coefficients were below 3%, with the lowest value (0.193%) observed for the 80 wt% ABS + 20 wt% TPU composition. This narrow statistical dispersion indicates stable printing conditions and good reproducibility of the tensile measurements. The reduction in tensile strength with increasing TPU content is consistent with TPU’s elastomeric nature, which has a lower modulus than both PLA and ABS. As the fraction of the soft phase increases, the load-bearing capacity becomes progressively governed by deformable TPU domains rather than the rigid thermoplastic matrix. Although this reduces peak stress, it also promotes greater strain accommodation during deformation.

**Figure 4:**
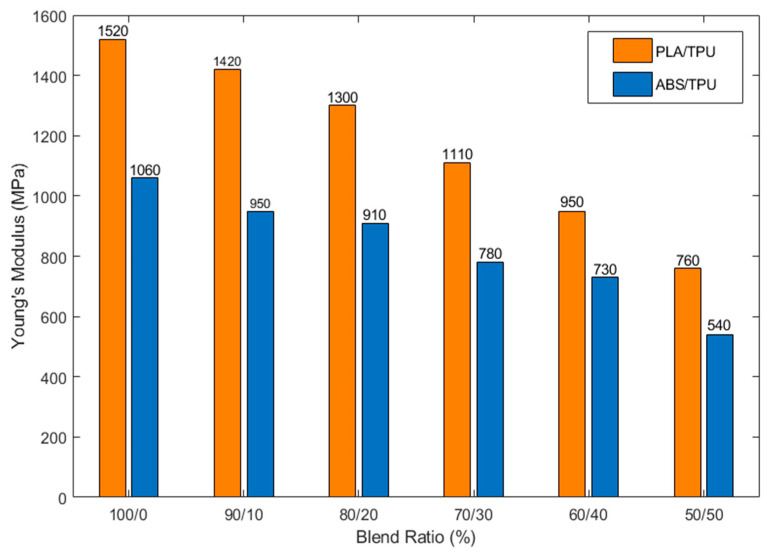
Young’s modulus values of PLA/TPU and ABS/TPU blends with varying TPU content.

A similar trend was observed for Young’s modulus (Figure 4). Neat PLA exhibited the highest stiffness (1520 MPa), while blends containing 50 wt% TPU showed nearly a 50% reduction in modulus. ABS-based systems had a lower initial modulus (1060 MPa for neat ABS) that softened progressively with increasing TPU content. This behavior reflects the growing contribution of the elastomeric phase, which reduces overall stiffness while enhancing material compliance and energy dissipation capability.

**Figure 5:**
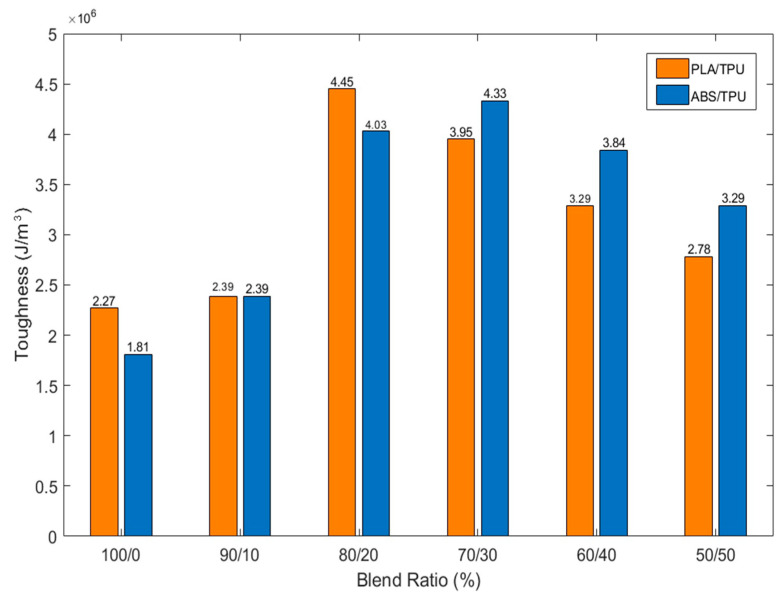
Toughness values of PLA/TPU and ABS/TPU blends with varying TPU content.

In contrast to tensile strength, toughness increased significantly with TPU incorporation (Figure 5). Neat PLA and ABS exhibited relatively low energy absorption capacities (2.27 and 1.81 J/m^3^, respectively). For PLA/TPU blends, toughness reached 4.45 J/m^3^ at 30 wt% TPU, corresponding to an improvement of approximately 96% relative to neat PLA. ABS/TPU blends showed an even stronger response, with toughness increasing to 4.03 J/m^3^ at 30 wt% TPU, representing an approximately 123% increase over neat ABS. At higher TPU contents (40–50 wt%), toughness values tended to plateau, remaining between 2.78 and 3.29 J/m^3^ for both material systems. This suggests that beyond a certain soft-phase fraction, additional TPU does not proportionally improve energy absorption and may instead begin to compromise structural integrity.

### 3.3. $\varepsilon_{yy}$ Strain Characterization via DIC

**Figure 6:**
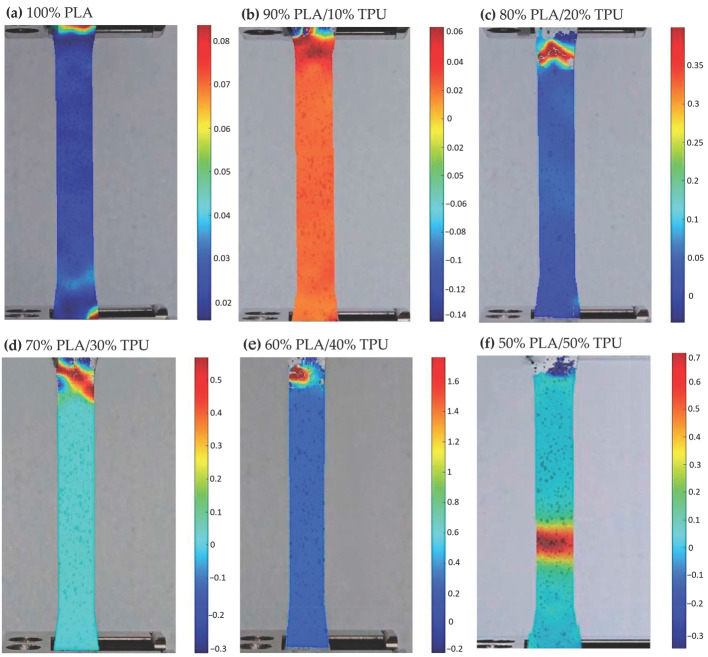
Full-field $\varepsilon_{yy}$ strain distributions of PLA/TPU tensile specimens obtained via DIC. Specimens include (**a**) 100% PLA, (**b**) 90% PLA/10% TPU, (**c**) 80% PLA/20% TPU, (**d**) 70% PLA/30% TPU, (**e**) 60% PLA/40% TPU, and (**f**) 50% PLA / 50% TPU. Each strain map represents the $\varepsilon_{yy}$ distribution at the maximum tensile deformation prior to failure.

**Figure 7:**
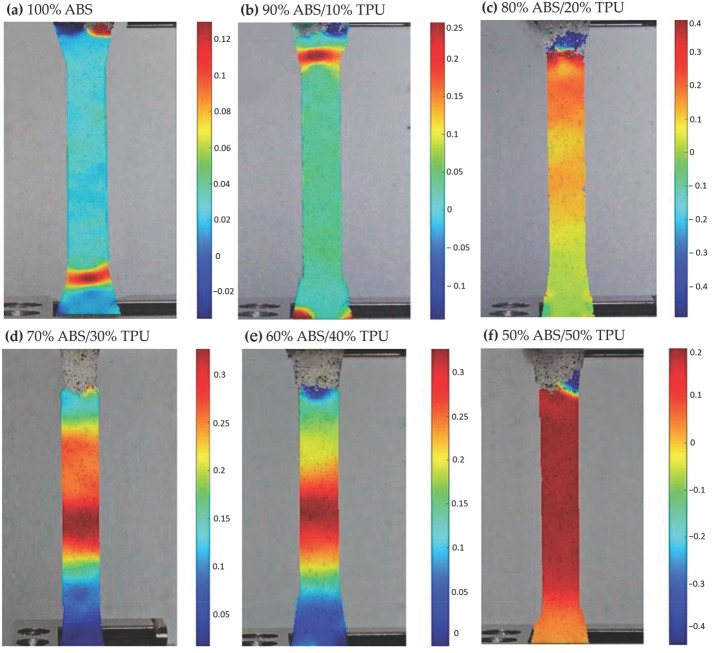
Full-field $\varepsilon_{yy}$ strain distributions of ABS/TPU tensile specimens obtained via DIC. Specimens include (**a**) 100% ABS, (**b**) 90% ABS/10% TPU, (**c**) 80% ABS/20% TPU, (**d**) 70% ABS/30% TPU, (**e**) 60% ABS/40% TPU, and (**f**) 50% ABS/50% TPU. The strain fields correspond to the $\varepsilon_{yy}$ component at the maximum tensile load and highlight regions of localized deformation. The color scales differ across images due to individual DIC analyses and automatic scaling by the Ncorr algorithm; therefore, strain magnitudes should be interpreted according to the color bar for each subfigure.

Full-field $\varepsilon_{yy}$ strain maps obtained from Digital Image Correlation (DIC) reveal clear differences in deformation behavior between PLA/TPU and ABS/TPU blends processed under identical conditions (PLA-based blends in Figure 6, ABS-based blends in Figure 7). Unlike conventional extensometry, DIC enables the visualization of localized strain accumulation, providing a more detailed interpretation of ductility evolution with increasing TPU content. For PLA-based specimens, neat PLA exhibited limited strain accommodation, with peak $\varepsilon_{yy}$ values slightly above 0.08. The addition of 10 wt% TPU did not significantly alter this behavior (≈0.06), suggesting that at low TPU content, the elastomeric phase does not substantially modify the dominant brittle deformation mode. A pronounced change was observed at 20 wt% TPU, where the peak strain increased to approximately 0.35, indicating the onset of more distributed deformation and improved strain transfer between phases. Maximum ductility within the PLA/TPU series was observed at 40 wt% TPU (≈1.60). At this composition, strain localization became more gradual and extended over a broader region of the gauge section, reflecting enhanced extensibility and energy absorption capacity. However, further increasing the TPU content to 50 wt% reduced the peak strain to approximately 0.70, suggesting that excessive soft-phase content may reduce effective load sharing within the blend. The ABS-based blends exhibited a different deformation trend. Neat ABS already displayed moderate strain capacity (≈0.12). The peak $\varepsilon_{yy}$ strain increased to approximately 0.40 at 20 wt% TPU, indicating a favorable balance between matrix stiffness and elastomeric contribution. At higher TPU contents (30–50 wt%), the peak strain gradually decreased (≈0.30 to 0.20), suggesting a progressive reduction in matrix continuity as the soft-phase fraction increased. Overall, the DIC results indicate composition-dependent optima for maximizing ductility: approximately 40 wt% TPU for PLA-based blends and around 20 wt% TPU for ABS-based blends. These strain maxima are consistent with the trends observed in tensile toughness measurements, reinforcing the relationship between blend composition and macroscopic deformation behavior. Specifically, the transition from localized brittle failure to more distributed plastic deformation, as captured by full-field DIC mapping, provides meaningful functional insight into the evolving deformation behavior of the blends. In PLA/TPU systems, the marked increase in peak $\varepsilon_{yy}$ strain at 20 wt% TPU (from 0.06 to 0.35) is consistent with the onset of a more effectively toughened response, likely associated with matrix yielding and shear deformation mechanisms commonly reported in rubber-modified polymers. Similarly, for ABS/TPU blends, the observed reduction in peak strain at higher TPU loadings (above 20 wt%), from approximately 0.40 to 0.20, is consistent with reduced matrix continuity and less efficient load transfer as the soft-phase fraction increases, in agreement with morphology–property relationships reported in the literature [5,11,36]. When interpreted together with DSC-confirmed changes in phase mobility, these dynamic strain distribution patterns provide a robust phenomenological basis for explaining the deformation behavior of the blends. Additional morphological characterization could further complement these findings, but the present integrated analysis already offers strong evidence for the proposed toughening trends. A summary of the peak $\varepsilon_{yy}$ strain values obtained from DIC analysis is presented in Table 5.

### 3.4. DIC and Tensile Elongation Comparison

**Figure 8:**
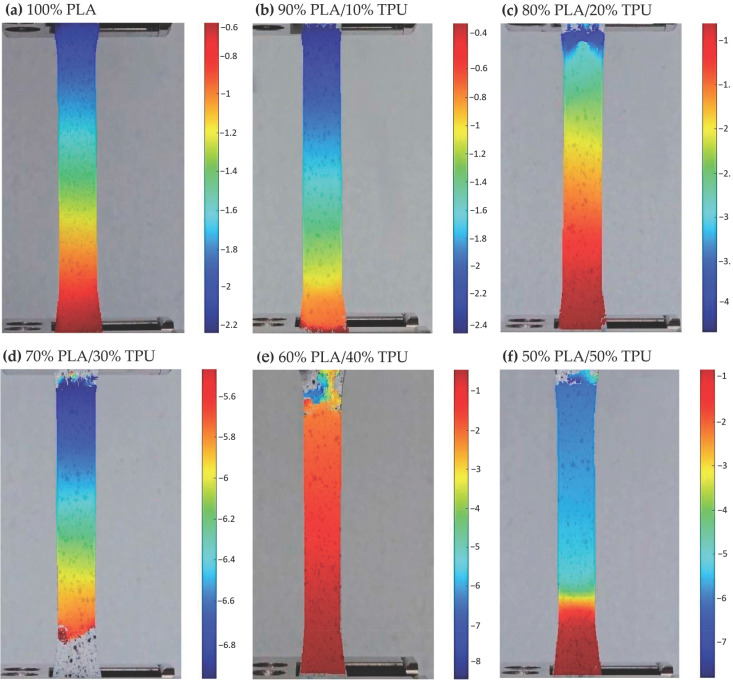
Full-field vertical displacement maps of PLA/TPU blends obtained via DIC. Subfigures (**a**–**f**) correspond to increasing TPU content: (**a**) 100% PLA, (**b**) 90% PLA/10% TPU, (**c**) 80% PLA/20% TPU, (**d**) 70% PLA/30% TPU, (**e**) 60% PLA/40% TPU, and (**f**) 50% PLA/50% TPU. Each map represents the total vertical displacement (mm) at the maximum tensile load. Color scales are individually adjusted by the Ncorr (v1.2) open-source DIC software operating on MATLAB R2024a (The MatWorks Inc.,USA) and therefore indicate the local elongation magnitude specific to each composition.

In addition to strain mapping, full-field vertical displacement data from the DIC analysis were used to quantify the overall elongation of specimens under tensile loading in both the PLA/TPU and ABS/TPU systems. For PLA/TPU blends, neat PLA exhibited a DIC displacement of 2.74 mm and a tensile elongation of 3.81 mm, whereas the 70% PLA / 30% TPU specimen showed the highest values, reaching 8.62 mm in DIC displacement and 9.90 mm in tensile testing. This behavior indicates an optimal balance between the elastomeric TPU phase and the rigid PLA matrix at this composition, as illustrated in Figure 8.

**Figure 9:**
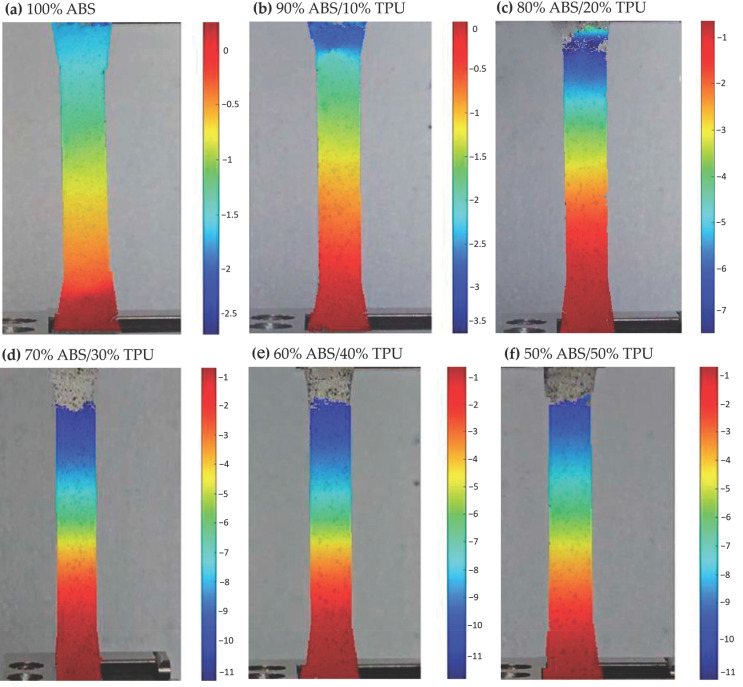
Full-field vertical displacement maps of ABS/TPU blends obtained via DIC. Subfigures represent increasing TPU content: (**a**) 100% ABS, (**b**) 90% ABS/10% TPU, (**c**) 80% ABS/20% TPU, (**d**) 70% ABS/30% TPU, (**e**) 60% ABS/40% TPU, and (**f**) 50% ABS/50% TPU. The maps illustrate the distribution of vertical displacement (mm) at the maximum tensile load. Individual color bars indicate the displacement range automatically scaled for each blend composition.

Similarly, in ABS/TPU blends, neat ABS showed the lowest displacement (2.60 mm) and elongation (4.37 mm). The 60% ABS/40% TPU blend exhibited the highest deformation levels, with 11.99 mm displacement measured by DIC and 15.11 mm elongation during tensile testing, highlighting the significant contribution of TPU to ductility enhancement in the ABS matrix (Figure 9).

Overall, the DIC-derived displacement measurements closely follow the elongation values obtained from conventional tensile testing while additionally capturing localized deformation fields that standard extensometers may not detect. This combined approach, therefore, provides a more comprehensive understanding of the deformation behavior of TPU-modified filaments.

The comparative DIC displacement and tensile elongation values are summarized in Table 6. This side-by-side comparison highlights the close agreement between full-field DIC measurements and conventional extensometer readings across all blend ratios, while also revealing localized deformation effects captured only by DIC. Notably, the 70% PLA/30% TPU and 60% ABS/40% TPU formulations exhibit the highest relative increases in displacement and elongation, confirming the optimal TPU contents identified in the strain-based analyses.

Notably, both PLA/TPU and ABS/TPU blends exhibited fracture within the gauge section at TPU contents up to 20 wt%. In contrast, at TPU contents of 30 wt% and higher, no complete fracture was observed under identical testing conditions. Consequently, the DIC-derived displacement shows a marked increase when transitioning from 20 wt% to 30 wt% TPU, indicating the onset of extensive plastic deformation beyond the conventional failure threshold.

### 3.5. DSC Thermal Behavior of PLA/TPU and ABS/TPU Blends

**Figure 10:**
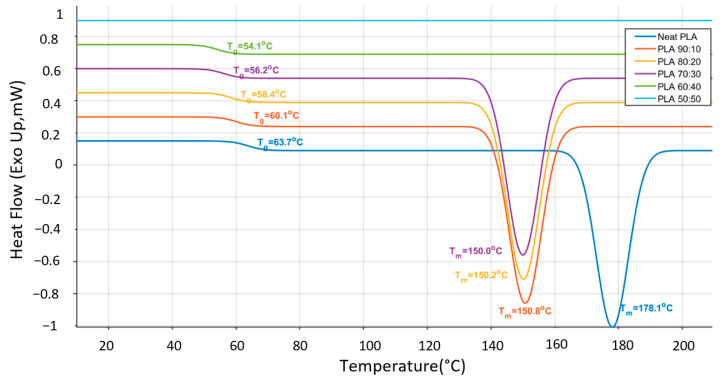
DSC thermograms of PLA/TPU blends showing the Tg and Tm transitions.

**Figure 11:**
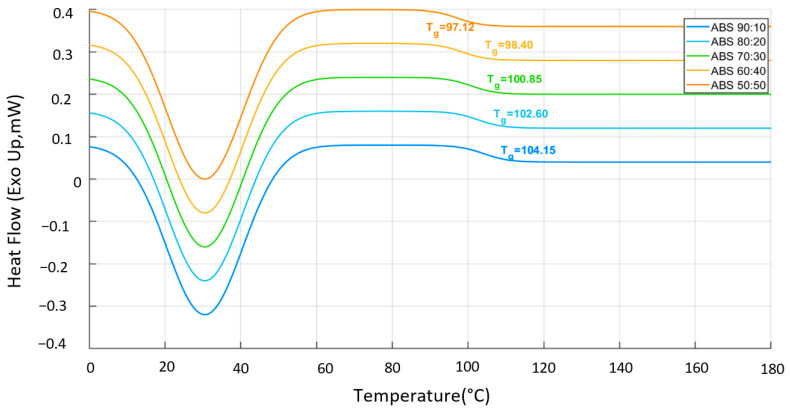
DSC thermograms of ABS/TPU blends indicating the Tg and broad thermal transitions.

The thermal behavior of PLA/TPU and ABS/TPU blends was investigated by differential scanning calorimetry (DSC), and the corresponding glass transition temperatures (Tg) and melting temperatures (Tm) are summarized in Table 7. Neat PLA exhibited a Tg of 63.71 °C and a Tm of 178.09 °C, consistent with the typical semi-crystalline thermal response reported for PLA materials [33,44]. With increasing TPU content, both Tg and Tm in the PLA-based blends gradually decreased up to 40 wt% TPU. This behavior suggests enhanced chain mobility and a progressive disruption of the crystalline phase, in agreement with previous studies on elastomer-modified PLA systems [8,31,38]. The reduction in the endothermic peak intensity observed in Figure 10 further supports a decrease in effective crystallinity as the elastomer fraction increases. For the 50/50 PLA/TPU blend, the glass transition Tg of the PLA phase could not be clearly resolved from the baseline due to the high elastomeric fraction of the TPU phase, which effectively suppressed the characteristic baseline shift; therefore, this value is reported as not detected (n.d.) in Table 7. For ABS-based systems, it should be noted that neat ABS is predominantly amorphous and therefore does not exhibit a true melting transition. Regarding the ABS/TPU blends, the Tg values were identified as broad and low-intensity transitions in the 95–110 °C range, which is consistent with the amorphous nature of the ABS matrix in Figure 11. Understanding these thermal transitions is crucial, as environmental factors and processing history can significantly influence the ductile-to-brittle transition in ABS-based materials [45]. Specifically, the previously indicated high-temperature endothermic features near 226 °C and 259 °C were re-evaluated using the raw DSC datasets. These signals were identified as instrumental artifacts occurring near the thermal stabilization limits of the DSC program rather than physical phase transitions. Similarly, the PLA/TPU series was re-analyzed to ensure methodological consistency across the entire study. The glass transition temperature (Tg) and melting temperature (Tm) of PLA-based blends were recalculated using the midpoint method, accurately reflecting the plasticization effect of the TPU phase. Furthermore, no Tm values are reported for the ABS series in Table 7, as the ABS matrix is inherently amorphous and does not exhibit a crystalline melting transition. While low-temperature endothermic peaks were recorded near 30 °C in these blends, they are attributed to the melting of the soft segments within the dispersed TPU phase rather than the ABS matrix itself. Consequently, Table 7 has been updated to reflect the accurate glass transition temperatures (e.g., 102.60 °C for the 80/20 blend) determined through midpoint analysis of the baseline shift. As the TPU fraction increased from 10 to 50 wt%, the Tg of the ABS phase showed a slight downward trend from 104.15 °C to 97.12 °C. This trend indicates a stronger influence of the elastomeric phase and increased segmental flexibility within the blend system, as the TPU acts as a physical plasticizer for the ABS chains. Reported Tg values for ABS generally fall within the range of 95–110 °C, depending on polymer grade and composition [46]. For the HI121H grade used in this study, manufacturer datasheets provide Vicat softening (93 °C) and heat deflection (79 °C) temperatures but do not specify Tg directly [33]. Consequently, Tg evaluation was primarily conducted for the ABS/TPU blends by identifying the midpoint of the characteristic baseline shifts. Overall, the DSC results demonstrate that TPU acts as an effective thermal modifier in both polymer systems, promoting increased molecular mobility and altering phase transition behavior. These findings are consistent with previous reports on thermoplastic elastomer–thermoplastic blends, in which TPU incorporation modifies crystallinity and broadens thermal transitions through partial phase interactions [13,38,46].

## 4. Discussion

The incorporation of TPU significantly alters the mechanical response and thermal behavior of PLA- and ABS-based systems. However, the magnitude and nature of these changes depend strongly on the host matrix structure. In PLA-based blends, the progressive reduction in tensile strength, accompanied by pronounced increases in elongation and toughness, reflects the well-known stiffness–ductility trade-off introduced by the elastomeric phases. However, the DIC strain analysis indicates that this transition does not occur uniformly across compositions. For PLA/TPU blends, two composition ranges appear particularly relevant. Around 20 wt% TPU, the blends exhibit the greatest improvement in energy absorption, whereas the maximum strain levels occur at approximately 40 wt% TPU. The separation between these two optima suggests that toughness enhancement and maximum ductility originate from different deformation mechanisms. At moderate TPU contents, the elastomeric phase likely improves stress redistribution within the PLA matrix and delays crack propagation. This behavior can be interpreted in terms of phase morphology, where dispersed TPU domains within the rigid PLA matrix act as local stress-relief regions. As the TPU fraction increases, the deformation mode shifts toward more distributed plastic deformation prior to failure. Nevertheless, beyond 40 wt% TPU, the strain capacity begins to decrease, indicating that excessive soft-phase content may reduce matrix continuity and limit effective load transfer within the blend structure. A different behavior is observed in ABS/TPU blends. In these systems, the maximum toughness and strain capacity occur at approximately 20–30 wt% TPU, after which further TPU addition progressively reduces both modulus and tensile strength. Compared with PLA, the predominantly amorphous ABS matrix appears to accommodate the elastomeric phase more readily, allowing mechanical optimization at relatively lower TPU contents. At higher TPU fractions, the mechanical response becomes increasingly dominated by the soft phase, leading to reduced structural rigidity and diminished load-bearing capacity. This earlier optimization may also be associated with more favorable phase interaction between ABS and TPU, which facilitates stress transfer at moderate elastomer contents. It should also be noted that the mechanical response of the specimens is influenced by the layer-by-layer architecture inherent to FDM fabrication. Interlayer bonding and printing-induced anisotropy can significantly affect crack initiation and propagation during tensile loading. The incorporation of TPU may partially mitigate brittle interlayer failure by enabling localized plastic deformation at the layer interfaces. This effect is consistent with the DIC strain maps, which reveal more distributed strain localization in TPU-containing blends compared with the highly localized deformation observed in neat polymers. It should be noted that the processing temperatures were deliberately optimized for each base polymer (210 °C for PLA and 240 °C for ABS) to ensure high-quality, defect-free samples, as using a single temperature for both systems would lead to either thermal degradation or incomplete extrusion. Regarding the deformation mechanisms, the DIC strain mapping provides critical phenomenological and macroscopic insights into how strain localizes and evolves during loading. The observed transition from localized brittle failure in pure “matrices to more distributed plastic deformation in 50:50 blends is consistent with well-established toughening mechanisms in rubber-modified polymer systems. While this macroscopic evidence highlights the role of TPU in promoting shear yielding and crazing, the reduction in peak strain at higher TPU concentrations in ABS-based systems may be attributed to phase continuity limits and altered load-transfer efficiency at the interface. These findings, supported by the existing morphology–property literature, suggest that the interfacial bonding achieved under the selected processing conditions is sufficient to facilitate significant energy dissipation during fracture. The thermal analysis further supports these mechanical observations. In PLA-based blends, the gradual decrease in Tg and Tm with increasing TPU content up to 40 wt% indicates enhanced molecular mobility and a progressive disruption of the crystalline structure. The absence of a clearly detectable glass transition temperature (Tg) in the 50:50 PLA/TPU blend is primarily attributed to the high content of Thermoplastic Polyurethane, which exhibits a broad and weak glass transition with a relatively small heat capacity change (ΔCp). As a result, the Tg signal becomes significantly attenuated and falls below the sensitivity limit of DSC measurements. In addition, the partial immiscibility between PLA and TPU leads to phase-separated morphologies, further suppressing the thermal transition signal and hindering its clear identification. In ABS-based systems, the absence of a distinct melting transition and the evolution of apparent thermal transitions with TPU addition are consistent with increased segmental mobility and partial phase interaction within the predominantly amorphous matrix. Therefore, the previously reported high-temperature transitions near 226 °C and 259 °C are not considered physical glass transitions, but rather instrumental artifacts associated with baseline fluctuations at elevated temperatures near the DSC’s heating limit. Specifically, the revised analysis of the ABS matrix, conducted using the midpoint Tmg method, demonstrates a controlled reduction in Tg from 104.15 °C to 97.12 °C as TPU content increases.

Overall, the results indicate the presence of matrix-dependent optimal composition windows rather than a universal TPU content. PLA/TPU blends exhibit maximum toughness at approximately 20 wt% TPU and maximum ductility at 40 wt% TPU, whereas ABS/TPU blends achieve a balanced mechanical response within the 20–30 wt% TPU range. From an application perspective, these findings provide practical guidance for designing TPU-modified filaments in material extrusion processes. By adjusting TPU content based on the underlying polymer architecture, it is possible to tailor the balance between stiffness, ductility, and energy absorption for applications that require controlled flexibility, impact resistance, or structural compliance in additively manufactured components.

## 5. Conclusions

This study systematically investigated the influence of TPU content (10–50 wt%) on the mechanical and thermal performance of PLA- and ABS-based filaments produced under consistent extrusion and FDM printing conditions. In both polymer systems, TPU acted as an effective ductility modifier, increasing strain capacity and energy absorption while simultaneously reducing stiffness and tensile strength.

For PLA-based blends, the most pronounced improvement in toughness occurred at 20 wt% TPU, whereas the highest ductility was achieved at 40 wt% TPU. The presence of these distinct optima indicates that maximizing energy absorption and maximizing elongation require different phase balances within the blend structure. At higher TPU contents (≥40–50 wt%), the increasing dominance of the elastomeric phase reduced structural rigidity and limited further improvements in mechanical performance. Notably, for the 50/50 PLA/TPU blend, the Tg transition of the PLA phase remained below the detection limit (n.d.) due to signal suppression by the high elastomer content.

ABS/TPU blends exhibited a different trend, achieving the most favorable balance between strength retention, toughness, and strain accommodation within the 20–30 wt% TPU range. Beyond this composition window, progressive softening and modulus reduction indicated increasing soft-phase dominance and a gradual loss of matrix continuity.

Full-field Digital Image Correlation (DIC) analysis provided additional insight into the deformation mechanisms of the blends. The DIC strain and displacement fields revealed a clear transition from localized brittle deformation in neat polymers toward more distributed plastic deformation with increasing TPU content. Furthermore, the close agreement between DIC-derived displacement measurements and conventional tensile elongation values confirms the reliability of full-field optical techniques for characterizing deformation behavior in additively manufactured polymer blends.

Process consistency was also verified through specimen mass measurements and statistical analysis of tensile data. The low coefficients of variation observed across all compositions indicate stable filament extrusion and reproducible FDM printing conditions, demonstrating that the measured mechanical trends primarily originate from material composition rather than geometric or manufacturing variability.

Thermal characterization by DSC supported these mechanical observations. The glass transition temperature Tg for the ABS/TPU blends, accurately determined by the midpoint method after discarding instrumental artifacts, was recorded in the 104.15 °C to 97.12 °C range. The gradual decrease in transition temperatures and the broadening of thermal events with increasing TPU content indicate enhanced molecular mobility and partial phase interaction within the blend systems. These thermal changes are consistent with the observed transition from rigid matrix-dominated behavior to elastomer-modified deformation mechanisms.

Overall, the results demonstrate that optimal TPU loading is matrix-dependent rather than universal. Approximately 20 wt% TPU maximizes toughness in PLA-based blends, 40 wt% TPU maximizes ductility in PLA systems, and 20–30 wt% TPU provides the most balanced mechanical response in ABS-based blends. These findings provide practical design guidelines for developing TPU-modified filaments for material extrusion processes that require a controlled balance between stiffness, ductility, and energy absorption.

Future study will extend this framework by incorporating azodicarbonamide (ADC) as a chemical foaming agent to produce lightweight, energy-absorbing PLA/TPU and ABS/TPU filaments for advanced FDM applications. While the present study provides a comprehensive understanding of the thermal and mechanical behavior of PLA/TPU blends, further spectroscopic investigations, such as Raman spectroscopy, could offer deeper insights into molecular-level interactions and interfacial bonding mechanisms between the PLA and Thermoplastic Polyurethane phases. Such analyses are planned for future studies, particularly in systems incorporating functional additives.

## Figures and Tables

**Table 1:** Supplier-reported properties of the raw materials.

Polymer	Density (g/cm^3^)	MFR (g/10 min)	Tensile Strength (MPa)	Elongation at Break (%)	Other Properties
ABS (HI121H)	1.05	22 (220 °C/10 kg)	50	>10	Vicat 93 °C
PLA (LX175)	1.24	6 (210 °C/2.16 kg)	45	≤5	Biobased, amorphous
TPU (140A90)	1.20	–	50	650	Shore A 90; Tear strength 140 N/mm; Abrasion 40 mm^3^

**Table 2:** Extrusion parameters for PLA/TPU and ABS/TPU blends.

Parameter	PLA/TPU Blends	ABS/TPU Blends
Barrel temperature profile (°C)	180–195	176–204
Cooling water bath (°C)	64	49
Haul-off speed (m·min^−1^)	21	28
Screw speed (rpm)	20	26.5
Die diameter (mm)	1.75	1.75
Single-Extruder diameter (mm)	30	30
Extruder L/D (mm)	24	24

**Table 3:** FDM printing parameters for tensile specimen fabrication.

Parameter	PLA/TPU Blends	ABS/TPU Blends
Layer height (mm)	0.2	0.2
Print speed (mm·s^−1^)	250	250
Filament diameter (mm)	1.75	1.75
Nozzle temperature (°C)	220	260
Bed temperature (°C)	65	95

**Table 4:** Mean ± standard deviation of specimen masses (*n* = 5) for PLA/TPU and ABS/TPU blends.

Blend Ratio (wt % TPU)	PLA/TPU Mass (g)	CV (%)	ABS/TPU Mass (g)	CV (%)
100/0	18.283 ± 0.058	0.32	15.284 ± 0.057	0.37
90/10	18.694 ± 0.052	0.28	14.857 ± 0.045	0.30
80/20	19.204 ± 0.026	0.14	15.934 ± 0.020	0.12
70/30	18.510 ± 0.050	0.27	15.446 ± 0.027	0.17
60/40	18.308 ± 0.019	0.10	15.778 ± 0.025	0.16
50/50	18.074 ± 0.037	0.21	16.061 ± 0.017	0.11

**Table 5:** Summary of Peak $\varepsilon_{yy}$ Strain values from DIC Analysis for PLA/TPU and ABS/TPU Blends.

TPU Content (wt %)	$\mathrm{PLA}\text{/}\mathrm{TPU}\;\mathrm{Peak}\;\varepsilon_{yy}$	$\mathrm{ABS}\text{/}\mathrm{TPU}\;\mathrm{Peak}\;\varepsilon_{yy}$
100	0.08	0.12
10/90	0.06	0.25
20/80	0.35	0.40
30/70	0.50	0.30
40/60	1.60	0.30
50/50	0.70	0.20

**Table 6:** Comparison of DIC-derived vertical displacement and tensile elongation for PLA/TPU and ABS/TPU Blends.

Blends (%)	DIC	Tensile Elegnation
100 ABS	2.6025	4.37
90/10	3.5304	5.48
80/20	8.7095	10.92
70/30	11.4592	15.02
60/40	11.9868	15.11
50/50	11.1137	15
100 PLA	2.7407	3.81
90/10	2.8591	4.24
80/20	4.50	9.88
70/30	8.616	9.9
60/40	8.459	9.88
50/50	8.2618	10.03

**Table 7:** The Tg and Tm of PLA/TPU and ABS/TPU blends obtained from DSC analyses.

Blends (%)	Tg (°C)	Tm (°C)
100 PLA	63.71	178.09
90/10	60.15	174.45
80/20	58.40	168.12
70/30	56.22	163.50
60/40	54.10	158.90
50/50	n.d.	-
100 ABS	105.2	-
90/10	104.15	-
80/20	102.6	-
70/30	100.85	-
60/40	98.40	-
50/50	97.12	-

n.d.: not detected. The glass transition temperature Tg for the 50/50 blend was not clearly discernible due to the significant suppression of the amorphous phase response by the high concentration of the elastomer (TPU) phase and the resulting overlap in thermal transitions.

## Data Availability

Data are available from the corresponding author upon reasonable request.

## Abbreviations

PLA	Polylactic acid
ABS	Acrylonitrile–butadiene–styrene
TPU	Thermoplastic polyurethane
DSC	Differential scanning calorimetry
DIC	Digital image correlation
FDM	Fused deposition modeling

## References

[1] Fang H., Zhang L., Chen A., Wu F. (2022). Improvement of Mechanical Property for PLA/TPU Blend by Adding PLA-TPU Copolymers Prepared via In Situ Ring-Opening Polymerization. Polymers.

[2] Jhao Y.-S., Ouyang H., Yang F., Lee S. (2022). Thermo-Mechanical and Creep Behaviour of Polylactic Acid/Thermoplastic Polyurethane Blends. Polymers.

[3] Sorimpuk N.P., Choong W.H., Chua B.-L. (2022). Thermoforming Characteristics of PLA/TPU Multi-Material Specimens Fabricated with Fused Deposition Modelling under Different Temperatures. Polymers.

[4] Thavornyutikarn B., Aumnate C., Kosorn W., Nampichai N., Janvikul W. (2023). Acrylonitrile Butadiene Styrene/Thermoplastic Polyurethane Blends for Material Extrusion Three-Dimensional Printing: Effects of Blend Composition on Printability and Properties. ACS Omega.

[5] Soltanmohammadi K., Rahmatabadi D., Aberoumand M., Soleyman E., Ghasemi I., Baniassadi M., Abrinia K., Baghani M. (2024). Effects of TPU on the mechanical properties, fracture toughness, morphology, and thermal analysis of 3D-printed ABS-TPU blends by FDM. J. Vinyl Addit. Technol..

[6] Shin E.J., Song Y.J., Jung Y.S., Jung I., Lee S. (2023). Manufacturing of Filament for 4D Printing through Polyether-Type TPU/PLA Blend. Adv. Polym. Technol..

[7] Raj A., Yousfi M., Prashantha K., Samuel C. (2024). Morphologies, Compatibilization and Properties of Immiscible PLA-Based Blends with Engineering Polymers: An Overview of Recent Works. Polymers.

[8] Kahraman Y., Özdemir B., Gümüş B.E., Nofar M. (2023). Morphological, rheological, and mechanical properties of PLA/TPU/nanoclay blends compatibilized with epoxy-based Joncryl chain extender. Colloid Polym. Sci..

[9] Żur A., Żur P., Michalski P., Baier A. (2022). Preliminary Study on Mechanical Aspects of 3D-Printed PLA-TPU Composites. Materials.

[10] Khatri N.R., Egan P.F. (2024). Energy Absorption of 3D Printed ABS and TPU Multimaterial Honeycomb Structures. 3D Print. Addit. Manuf..

[11] Rahmatabadi D., Ghasemi I., Baniassadi M., Abrinia K., Baghani M. (2022). 3D printing of PLA-TPU with different component ratios: Fracture toughness, mechanical properties, and morphology. J. Mater. Res. Technol..

[12] Abdelwahab M.A., Elkholy H.M., Khan A., Aayanifard Z., Wauldron N., Matuana L.M., Auras R., Juncheng Z., Cheng S., Rabnawaz M. (2024). Glycidol-Free Aliphatic Copolymers as Chain Extenders for Polylactic Acid and Their Cost and Carbon Emission Assessments. ACS Sustain. Chem. Eng..

[13] Hang Z., Lv Z., Feng L., Liu B. (2022). Study of Compatibility and Flame Retardancy of TPU/PLA Composites. Materials.

[14] Kuleyin H., Budak S., Yasan Ö.B., Gümrük R. (2025). Characterization of thermal, chemical, mechanical, and fatigue behavior of 3D printed ABS-based elastomeric blends: ABS/EVA and ABS/TPU. Polym. Test..

[15] Nejatpour M., Fallah A., Koc B. (2025). Shape Memory PLA/TPU Blend Using High-Speed Thermo-Kinetic Mixing. ACS Omega.

[16] Hamidi M.N., Abdullah J., Mahmud A.S., Hassan M.H., Zainoddin A.Y. (2025). Influence of thermoplastic polyurethane (TPU) and printing parameters on the thermal and mechanical performance of polylactic acid (PLA)/thermoplastic polyurethane (TPU) polymer. Polym. Test..

[17] Alonso A., Lázaro M., Lázaro D., Alvear D. (2023). Thermal characterization of acrylonitrile butadiene styrene-ABS obtained with different manufacturing processes. J. Therm. Anal. Calorim..

[18] Ujfalusi Z., Pentek A., Told R., Schiffer A., Nyitrai M., Maroti P. (2020). Detailed Thermal Characterization of Acrylonitrile Butadiene Styrene and Polylactic Acid Based Carbon Composites Used in Additive Manufacturing. Polymers.

[19] Refat M., Maertens R., Weiss P., Henning F., Schulze V., Liebig W.V. (2025). Investigation of the Influence of Manufacturing on Filament Production and Its Impact on Additive Manufactured Structures. Polymers.

[20] Yurtbasi Z., Cinar A.H., Sarisoy F., Kasgoz A. (2025). Effect of TPU Type and Concentration on the Rheological, Mechanical, and 3D Printing-Related Properties of PLA/TPU Blends. J. Appl. Polym. Sci..

[21] Guessasma S., Nouri H., Belhabib S. (2022). Digital Image Correlation and Finite Element Computation to Reveal Mechanical Anisotropy in 3D Printing of Polymers. Materials.

[22] Mousa M.A., Yussof M.M., Hussein T.S., Assi L.N., Ghahari S. (2023). A Digital Image Correlation Technique for Laboratory Structural Tests and Applications: A Systematic Literature Review. Sensors.

[23] Atahan M.G., Sevim C., Demirbas M.D., Apalak M.K. (2025). Comparative study on bending performances of 3D-printed monolithic and adhesively bonded sandwich structures with various auxetic cores: An innovative production approach. J. Sandw. Struct. Mater..

[24] Ravago Petrokimya (2023). Ravathane^®^ 140 A90 Thermoplastic Polyurethane—Technical Data Sheet.

[25] Lim C., Jeong Y., Limkantanyu S., Kwon M. (2022). Strain Measuring of Composite Grid Using Digital Image Correlation. Adv. Mater. Sci. Eng..

[26] Melinda A.P., Higuchi S., Yoresta F.S., Yamazaki Y., Nhut P.V., Nuryanti P., Takiuchi Y., Matsumoto Y. (2025). Digital image correlation (DIC) application to evaluate bending performance of timber beams strengthened with NSM-CFRP plate. Eur. J. Wood Prod..

[27] Rahmatabadi D., Soltanmohammadi K., Pahlavani M., Aberoumand M., Soleyman E., Ghasemi I., Baniassadi M., Abrinia K., Bodaghi M., Baghani M. (2023). Shape memory performance assessment of FDM 3D printed PLA-TPU composites by Box-Behnken response surface methodology. Int. J. Adv. Manuf. Technol..

[28] Cetiner B., Sahin Dundar G., Yusufoglu Y., Saner Okan B. (2023). Sustainable Engineered Design and Scalable Manufacturing of Upcycled Graphene Reinforced Polylactic Acid/Polyurethane Blend Composites Having Shape Memory Behavior. Polymers.

[29] Abidaryan S., Akhoundi B., Hajami F. (2023). Additive manufacturing and investigation of shape memory properties of polylactic acid/thermoplastic polyurethane blend. J. Elastomers Plast..

[30] Dezianian S., Azadi M. (2023). Multi-material metamaterial topology optimization to minimize compliance and weight constraints in PLA/TPU additive manufacturing. Polymers.

[31] Fereydoonpour F., Dezianian S., Azadi M. (2025). Shape memory recovery in polylactic acid and thermoplastic polyurethane bi-material metamaterials fabricated by additive manufacturing under fatigue testing. Polym. Test..

[32] Palabıyık E., Tekay E. (2022). Design and characterization of TPU-based blends for heat-triggered shape memory applications. Polym. Adv. Technol..

[33] LG Chem (2024). ABS HI121H Technical Data Sheet.

[34] TotalEnergies Corbion (2022). Luminy^®^ LX175 PLA Product Data Sheet.

[35] Santus C., Neri P., Romoli L., Cococcioni M. (2024). Residual Stress Determination with the Hole-Drilling Method on FDM 3D-Printed Precurved Specimen through Digital Image Correlation. Appl. Sci..

[36] (2014). Standard Test Method for Tensile Properties of Plastics.

[37] Vidakis N., Petousis M., Mountakis N., David C.N., Sagris D., Das S.C. (2023). Thermomechanical response of thermoplastic polyurethane used in MEX additive manufacturing over repetitive mechanical recycling courses. Polym. Degrad. Stab..

[38] Plamadiala I., Croitoru C., Pop M.A., Roata I.C. (2025). Enhancing Polylactic Acid (PLA) Performance: A Review of Additives in Fused Deposition Modelling (FDM) Filaments. Polymers.

[39] Darnal A., Shahid Z., Deshpande H., Kim J., Muliana A. (2023). Tuning mechanical properties of 3D printed composites with PLA:TPU programmable filaments. Compos. Struct..

[40] Ncorr Open-Source DIC Software. https://www.ncorr.com/index.php/downloads.

[41] Yu W., Li M., Lei W., Chen Y. (2024). FDM 3D Printing and Properties of PBAT/PLA Blends. Polymers.

[42] Jayswal A., Adanur S. (2023). Characterization of polylactic acid/thermoplastic polyurethane composite filaments manufactured for additive manufacturing with fused deposition modeling. J. Thermoplast. Compos. Mater..

[43] Holmes J., Sommacal S., Das R., Stachurski Z., Compston P. (2023). Digital image and volume correlation for deformation and damage characterisation of fibre-reinforced composites: A review. Compos. Struct..

[44] Rigotti D., Dorigato A., Pegoretti A. (2024). Multifunctional 3D-Printed Thermoplastic Polyurethane (TPU)/Multiwalled Carbon Nanotube (MWCNT) Nanocomposites for Thermal Management Applications. Appl. Sci..

[45] Freymond C., Mackré-Delannoy X., Guinault A., Charbuillet C., Fayolle B. (2022). Thermal oxidation of acrylonitrile-butadiene-styrene: Origin of the ductile/brittle transition. Polym. Degrad. Stab..

[46] Wang Z., Wang L., Tang F., Shen C. (2024). PLA-Based Composite Panels Prepared via Multi-Material Fused Filament Fabrication and Associated Investigation of Process Parameters on Flexural Properties of the Fabricated Composite. Polymers.

